# Prognostic Impact of Adjuvant Immunotherapy in Patients with High-Risk Upper Tract Urothelial Cancer: Results from the ROBUUST 2.0 Collaborative Group

**DOI:** 10.3390/cancers17132144

**Published:** 2025-06-25

**Authors:** Maxwell Otiato, Farshad Sheybaee Moghaddam, Alireza Ghoreifi, Riccardo Autorino, Gabriele Bignante, Chandru Sundaram, Daniel Sidhom, Ithaar H. Derweesh, Dhruv Puri, Vitaly Margulis, Benjamin Popokh, Firas Abdollah, Alex Stephens, Matteo Ferro, Giuseppe Simone, Gabriele Tuderti, Reza Mehrazin, Ahmed Eraky, Mark Gonzalgo, Omar Falik Nativ, Zhenjie Wu, Francesco Porpiglia, Enrico N. Checcucci, Andres Correa, Randall Lee, Alessandro Antonelli, Alessandro Veccia, Soroush Rais-Bahrami, Alireza Dehghanmanshadi, Nirmish Singla, Stephan Brönimann, Sisto Perdonà, Roberto Contieri, Takashi Yoshida, James Porter, Saum Ghodoussipour, Luca Lambertini, Andrea Minervini, Hooman Djaladat

**Affiliations:** 1 Institute of Urology, University of Southern California, 1441 Eastlake Ave, Suite 7416, Los Angeles, CA 90089, USA; otiato@usc.edu (M.O.); farshad.sheybaeemoghaddam@med.usc.edu (F.S.M.); alireza.ghoreifi@med.usc.edu (A.G.); 2 Department of Urology, Rush University, Chicago, IL 60612, USA; riccardo_autorino@rush.edu (R.A.); gabriele.bignante@unito.it (G.B.); 3 Department of Urology, Indiana University, Indianapolis, IN 47405, USA; sundaram@iupui.edu (C.S.); sidhom@iupui.edu (D.S.); 4 Department of Urology, UC San Diego School of Medicine, La Jolla, CA 92093, USA; iderweesh@ucsd.edu (I.H.D.); dhruvpuri1997@gmail.com (D.P.); 5 Department of Urology, University of Texas Southwestern Medical Center, Dallas, TX 75390, USA; vitaly.margulis@utsouthwestern.edu (V.M.); benjamin.popokh@utsouthwestern.edu (B.P.); 6 Vattikuti Urology Institute, Henry Ford Hospital, Detroit, MI 48202, USA; firas.abdollah@gmail.com (F.A.); alexstephens@hfhs.org (A.S.); 7 Unit of Urology, Department of Health Science, University of Milan, ASST Santi Paolo e Carlo, 20172 Milan, Italy; drmatteoferro@gmail.com; 8 Department of Urology, IRCCS ''Regina Elena'' National Cancer Institute, 00144 Rome, Italy; puldet@gmail.com (G.S.); gabriele.tuderti@gmail.com (G.T.); 9 Department of Urology, Icahn School of Medicine at Mount Sinai Hospital, New York, NY 10029, USA; reza.mehrazin@gmail.com (R.M.); ahmed.eraky@mountsinai.org (A.E.); 10 Desai Sethi Urology Institute, University of Miami Miller School of Medicine, Miami, FL 33136, USA; m.gonzalgo@med.miami.edu (M.G.); nativ@miami.edu (O.F.N.); 11 Department of Urology, Changhai Hospital, Naval Medical University, Shanghai 200433, China; wuzhenjie17@163.com; 12 Department of Surgery, Candiolo Cancer Institute, FPO-IRCCS, 10060 Candiolo, Italy; porpiglia@libero.it (F.P.); checcu.e@hotmail.it (E.N.C.); 13 Fox Chase Cancer Center, Philadelphia, PA 19111, USA; andres.correa@fccc.edu (A.C.); randall.lee@fccc.edu (R.L.); 14 Department of Urology, University of Verona, 37129 Verona, Italy; alessandro_antonelli@me.com (A.A.); a.veccia88@gmail.com (A.V.); 15 Department of Urology, University of Alabama at Birmingham Heersink School of Medicine, Birmingham, AL 35294, USA; soroush@uab.edu (S.R.-B.); adehghanmanshadi@uabmc.edu (A.D.); 16 The James Buchanan Brady Urological Institute at John Hopkins, Baltimore, MD 21205, USA; nsingla2@jhmi.edu (N.S.); sbronim1@jh.edu (S.B.); 17 Department of Urology, Istituto Nazionale Tumori “Fondazione Pascale”, 80131 Naples, Italy; s.perdona@istitutotumori.na.it (S.P.); contieri.ro@gmail.com (R.C.); 18 Department of Urology and Andrology, Kansai Medical University, Osaka 5708507, Japan; palmfiz2009@gmail.com; 19 Swedish Medical Center, Seattle, WA 98122, USA; porter@swedishurology.com; 20 Section of Urologic Oncology, Rutgers Cancer Institute, Rutgers Robert Wood Johnson Medical School, New Brunswick, NJ 08901, USA; sg1621@cinj.rutgers.edu; 21 Oncologic Minimally Invasive Urology and Andrology Unit, Careggi Hospital, University of Florence, 50134 Florence, Italy; luca.lambertini@unifi.it (L.L.); andrea.minervini@unifi.it (A.M.)

**Keywords:** upper tract urothelial carcinoma, immunotherapy, outcomes, nephroureterectomy

## Abstract

**Background/Objective**: The impact of adjuvant immunotherapy (IO) on the prognosis of patients with upper tract urothelial carcinoma (UTUC) remains unclear. This study examines the association of adjuvant IO with oncologic outcomes in patients with high-risk UTUC. **Methods**: This retrospective study reviewed patients with high-risk UTUC treated with adjuvant IO using the ROBotic surgery for Upper tract Urothelial cancer STudy (ROBUUST) database. Propensity-score-matched analysis (nearest-neighbor algorithm, caliper 0.1) was conducted to compare patients receiving adjuvant IO versus those who did not, with matching based on pathologic T and N category and receipt of neoadjuvant chemotherapy. Associations between adjuvant IO and urothelial recurrence-free survival (URFS), non-urothelial recurrence-free survival (NRFS), and overall survival (OS) were estimated using a Cox proportional hazards model. **Results**: Seventy-five patients received adjuvant IO following nephroureterectomy (median four cycles, including eleven (14.7%) nivolumab, thirty-one (41.3%) pembrolizumab, four (5.3%) atezolizumab, and twenty-nine (38.6%) other agents. These patients were matched to 68 patients without adjuvant therapy. Median follow-up times were 17 (IQR, 10–29) months and 20 (9–44) months for IO and no adjuvant therapy, respectively. Multivariable analysis revealed that adjuvant IO was not associated with URFS, NRFS, or OS. Pathologic nodal involvement (HR 7.52, *p* < 0.001) was the only independent predictor of worse OS. **Conclusions**: In this real-world retrospective data set, adjuvant IO does not have an impact on oncologic outcomes of UTUC patients following extirpative surgery.

## Simple Summary

This study investigated the effect of immunotherapy on outcomes in patients with high-risk upper tract urothelial carcinoma (UTUC) following surgery. Using a large multi-institutional database, outcomes were compared between patients treated with immunotherapy and a matched group who received no additional therapy. Matching was based on tumor category, lymph node involvement, and prior chemotherapy. Results showed no significant improvement in recurrence-free or overall survival with immunotherapy. However, the presence of cancer in lymph nodes remained a strong predictor of poor survival. These findings suggest that immunotherapy may not provide added benefit in the current setting and highlight the need for better risk-based treatment strategies in high-risk UTUC.

## 1. Introduction

Upper tract urothelial carcinoma (UTUC) accounts for 5–10% of all urothelial tumors [1,2]. While radical nephroureterectomy (RNU) remains the standard curative treatment for localized and high-risk UTUC, surgical resection overall remains the only curative option for localized UTUC [3]. Adjuvant systemic therapy is currently recommended for high-stage UTUC patients following surgery [4,5]. Recent trials investigating adjuvant systemic chemotherapy in high-risk UTUC have continued to demonstrate improved outcomes [6].

A breakthrough in the discovery and adoption of immunotherapy (IO), targeted agents, and antibody–drug conjugates (ADCs) has transformed the systemic treatments of urothelial cancers across the disease continuum, including non-muscle-invasive bladder cancer (NMIBC) and muscle-invasive bladder cancer (MIBC). For NMIBC, intravesical Bacillus Calmette–Guérin (BCG) remains the gold standard for patients with high-risk disease. In cases unresponsive to BCG, pembrolizumab has been approved and has demonstrated efficacy, as highlighted in the KEYNOTE-057 trial. Additionally, emerging approaches, including novel intravesical gene therapies and combination intravesical chemotherapies, have shown promising early results [7,8,9]. For MIBC, neoadjuvant cisplatin-based chemotherapy followed by radical cystectomy remains standard, with immune checkpoint inhibitors now being incorporated into perioperative treatment settings. For metastatic disease, platinum-based chemotherapy continues to serve as the first-line treatment; however, the combination of enfortumab vedotin and pembrolizumab has brought forth unprecedented survival benefits, offering great promise to a new standard of care. ADCs such as enfortumab vedotin and sacituzumab govitecan, along with FGFR inhibitors like erdafitinib for tumors with FGFR2/3 alterations, are now established as later lines of therapy [9,10,11,12,13,14,15].

For UTUC, the role of immunotherapy is still emerging. Currently, adjuvant nivolumab therapy is an option for patients with UTUC who have undergone neoadjuvant platinum-based chemotherapy or for those who are ineligible or refuse perioperative cisplatin [7,16]. The results of the CheckMate 274 trial showed adjuvant nivolumab significantly improves disease-free survival (DFS) compared to placebo in patients with locally advanced urothelial carcinoma after cystectomy or nephroureterectomy [17,18]. Similarly, the AMBASSADOR trial demonstrated that adjuvant pembrolizumab significantly improves DFS in patients with high-risk muscle-invasive urothelial carcinoma (MIUC), including those with UTUC, after radical surgery [19]. In contrast, the IMvigor 010 study found that adjuvant atezolizumab did not significantly enhance DFS compared to observation in patients with high-risk MIUC, also including those with UTUC [13]. While current literature provides evidence for MIUC, powered evidence specifically evaluating the role of adjuvant IO therapies in UTUC remains limited. Real-world data may offer valuable insights into the effectiveness and safety of adjuvant IO in UTUC, especially given the rarity of the disease and the limited availability of randomized trials. The impact of other adjuvant IOs on prognosis and survival in UTUC patients remains understudied.

This study aims to examine the association between adjuvant IO and oncological outcomes in patients with high-risk UTUC, evaluate clinical and pathological factors and treatment patterns as predictors of response to adjuvant IO, and offer perspectives into the role of adjuvant IO and its integration into existing management protocols for high-risk UTUC.

## 2. Materials and Methods

This retrospective cohort study utilized data from the ROBotic surgery for Upper Tract Urothelial cancer STudy (ROBUUST) database, a multi-institutional registry of patients undergoing surgery for UTUC across 17 centers worldwide. Data-sharing agreements were established with each center and Institutional Review Board (IRB) approval was obtained at all participating centers (IRB No. 161197). The inclusion criteria were patients with high-risk UTUC, according to European Association of Urology guidelines, who underwent curative robotic nephroureterectomy or segmental ureterectomy and were treated with adjuvant IO between January 2015 and December 2022 [20]. Patients with unknown pathologic stage, unknown receipt of adjuvant IO, and missing survival data were excluded. Four groups were identified based on adjuvant therapy use: IO, chemotherapy, a combination of IO and chemotherapy, and no adjuvant therapy. Of note, patients received adjuvant systemic therapy according to each center’s multidisciplinary decision.

The study collected data on demographic, clinicopathological, pathological, and survival variables. Demographic and baseline characteristics included age, gender, body mass index (BMI), American Society of Anesthesiologists (ASA) score, Eastern Cooperative Oncology Group (ECOG) status, history of type 2 diabetes mellitus, history of bladder cancer, tumor size, tumor location, and type of surgery. Pathological variables consisted of pathological TNM staging, grade, tumor necrosis, lymphovascular invasion, tumor multifocality, and margin status.

The primary outcome, urothelial recurrence-free survival (URFS), was calculated from the date of surgery to the date of first documented clinical recurrent disease in the bladder, contralateral ureter, or contralateral renal pelvis, as diagnosed by biopsy. If the patient did not have recurrence documented, the outcome was calculated as the date of last follow-up or death, indicating the patient was considered recurrence-free up until that time. Secondary outcomes included non-urothelial recurrence-free survival (NRFS) and overall survival (OS). NRFS was defined as survival without recurrent disease identified by clinical or paraclinical investigations including imaging outside the bladder or the contralateral upper tract.

Data analysis was conducted using IBM SPSS v25 (IBM Corp, Armonk, NY, USA), with statistical significance defined as *p* < 0.05. Baseline characteristics of treatment groups were compared using χ^2^ tests for categorical variables and ANOVA for continuous variables. Categorical variables were reported as frequencies and percentages, while continuous variables were presented as medians with interquartile ranges (IQRs).

Propensity scores were estimated using logistic regression models that included pathological T and N category and the receipt of neoadjuvant chemotherapy. The IO group was matched to the no adjuvant therapy group using a 1:1 nearest-neighbor algorithm without replacement (caliper width = 0.1 SD).

URFS, NRFS, and OS were compared between the IO group and the no adjuvant therapy group using an adjusted Kaplan–Meier method. A multivariable Cox proportional hazards regression model was performed to assess baseline and pathological variables as independent factors associated with survival outcomes.

## 3. Results

Among the 1911 patients initially included in the ROBUUST registry, 219 (11%) patients received adjuvant chemotherapy, 27 (1.4%) patients received a combination of chemotherapy and IO, and 1590 (83%) patients were not treated with any systemic therapy. A total of 75 (3.9%) patients received IO alone (mean (IQR) age, 73 (67–79) years; 65% male). Pembrolizumab was the most common type of adjuvant IO (31 patients (41.3%)), followed by nivolumab (13 patients (17.3%)), atezolizumab and avelumab (2 patients (2.67%) each), and durvalumab (1 patient (1.3%)). The remaining 25 patients (33.3%) received an unspecified immunotherapy.

Baseline and clinical features (before and after propensity score matching (PSM)) for the treatment groups are shown in Table 1. Based on PSM including pathological T and N category and the receipt of neoadjuvant chemotherapy, there were 75 patients who received adjuvant IO and 68 patients who did not receive adjuvant therapy. The median time to follow-up was 17 (IQR, 10–29) months and 20 (9–44) months for the IO and no adjuvant therapy groups, respectively. There were no significant differences in demographic characteristics or pathologic features between the two groups. In the IO cohort, 51 patients (69.9%) had pathologic T category greater than T2, 45 patients (61.6%) had multifocal tumors, and 37 patients (50.7%) had received prior neoadjuvant therapy. Regarding nodal status, 26 patients (36.6%) were classified as pN0, while 19 patients (26.8%) and 26 patients (36.6%) were classified as pN+ and pNx, respectively (Table 1).

**Figure 1:**
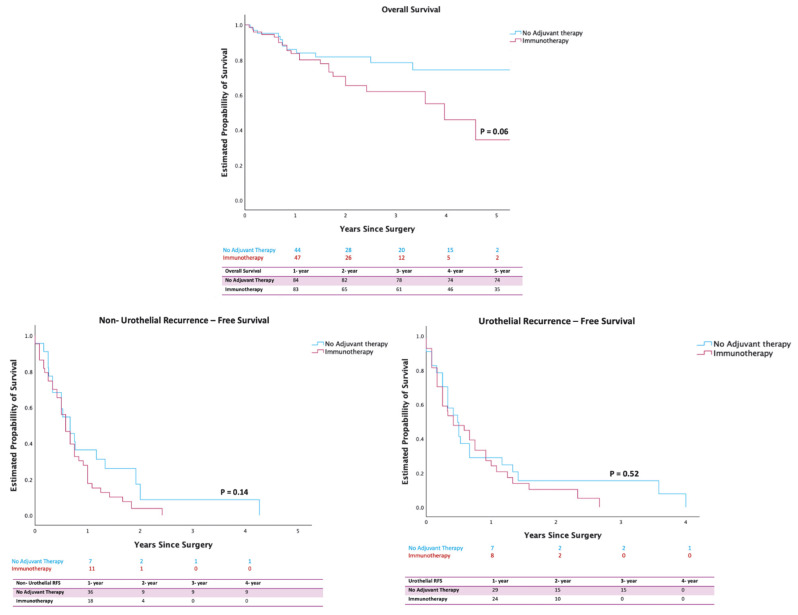
Kaplan–Meier Analysis of Estimated Probability for Oncologic Outcomes.

The OS rate at 1 year for the adjuvant IO vs. non-IO groups was 83% vs. 84% (*p* = 0.06), indicating no significant difference. Similarly, the URFS rate at 1 year for the adjuvant IO vs. non-IO groups was 24% vs. 29% (*p* = 0.52), showing no significant difference. Compared with the cohort with no adjuvant therapy, the cohort with IO had a higher proportion of non-urothelial recurrences observed within 9 months after PSM (IO, 41 (54.7%); no adjuvant therapy, 20 (31.3%); *p* = 0.006). However, when time-to-event was accounted for using Kaplan–Meier analysis, the 1-year survival probability was 18% vs. 30% for the adjuvant IO and non-IO groups (*p* = 0.14), with no detected differences (Figure 1).

**Figure 2:**
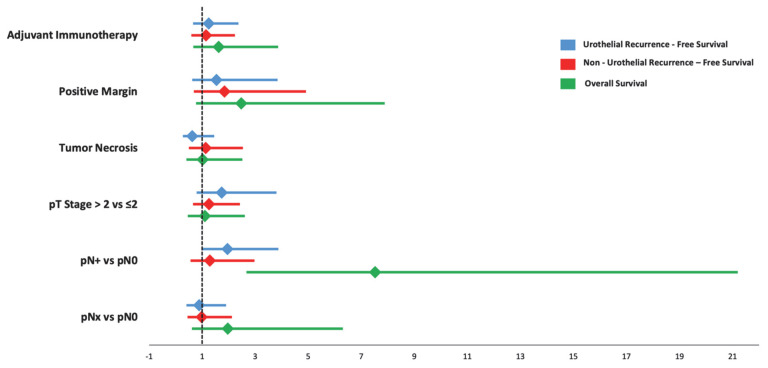
Multivariable Cox Regression Forest Plot of Variables Associated with Oncologic Outcomes.

The presence of pathologic nodal disease (pN+) was associated with significantly worse OS (HR, 7.52; 95% CI, 2.67–21.2) following multivariable Cox regression analysis. No other pathological factors were found to be independent predictors of URFS, NRFS, and OS (Figure 2).

## 4. Discussion

This retrospective study evaluated the association between IO and survival outcomes among patients with UTUC following surgery with curative intent from centers worldwide. The diverse cohort study of data found no evidence of a survival or oncologic benefit from adjuvant IO in this patient population.

While the adjuvant IO group demonstrated a higher proportion of non-urothelial recurrence within the study period, Kaplan–Meier analysis did not detect a statistically significant difference in NRFS between groups at 1 year. The greater number of recurrences in the IO group (54.7% vs. 31.3%) may reflect clustering of early events in our cohort. However, survival analysis revealed that overall recurrence risk over time was not significantly different. Our results suggest that, while IO-treated patients may experience earlier non-urothelial recurrence, their risk of long-term recurrence is not statistically different from those who received no adjuvant therapy.

In recent years, indications for adjuvant IO in patients with urothelial cancer have been evolving, driven by an increasing number of studies [21,22]. However, research dedicated to the investigation of this topic in UTUC remains limited, with most powered evidence derived from studies on locally advanced or metastatic urothelial carcinoma of the bladder. This may be due in part to the rarity of UTUC and the heterogeneity of the disease, making it difficult to conduct large-scale, UTUC-specific trials with adjuvant immunotherapies beyond nivolumab, pembrolizumab, and atezolizumab [23].

Current evidence from trials presents mixed findings regarding the evolving role of immunotherapy in UTUC [16]. The CheckMate 274 trial demonstrated a median DFS of 20.8 months with nivolumab, compared to 10.8 months with placebo (HR, 0.70; 98.22% CI, 0.55–0.90; *p* < 0.001), in patients with high-risk MUIC. The expanded analysis confirmed DFS benefits across various subgroups, including UTUC, and highlighted that adjuvant nivolumab significantly improves DFS, particularly among patients with higher PD-L1 expression [17,18].

Similarly, the AMBASSADOR trial, which compared adjuvant pembrolizumab to observation in patients with high-risk MUIC, reported a median DFS of 29.6 months with pembrolizumab compared to 14.2 months with observation (HR, 0.73; 95% CI, 0.59–0.90; *p* = 0.003), indicating adjuvant pembrolizumab significantly confers a DFS advantage for patients, including patients with UTUC [18]. However, a systematic review and meta-analysis by Sayyid et al. showed no observed DFS benefit in patients with UTUC when treated with pembrolizumab and other adjuvant immune checkpoint inhibitors (HR, 1.19; 95% CI, 0.86–1.64) [24]. The differences in disease-free survival outcomes between this trial and our study may be attributed to variations in patient selection, trial design, biomarker stratification, and duration of follow-up.

The IMvigor 010 trial, which randomized patients with postoperative MUIC to receive either adjuvant atezolizumab or observation, found no significant DFS improvement (19.4 vs. 16.6 months; HR, 0.89; 95% CI, 0.74–1.08; *p* = 0.24). The study also found adverse events were more frequent in the atezolizumab group, with serious adverse events occurring in 31% of patients compared to 18% in the observation group [25].

Additionally, a comprehensive meta-analysis by Laukhtina et al. reported that adjuvant chemotherapy is associated with a significantly lower likelihood of disease progression in UTUC than observation/placebo [26]. IOs, such as atezolizumab and nivolumab, did not demonstrate a similar benefit but had a comparable risk of adverse effects to that of the observation/placebo group. These findings support the use of adjuvant chemotherapy over IOs in the treatment of high-risk UTUC following extirpative surgery and align with results from the POUT trial, which showed DFS benefit with adjuvant platinum-based chemotherapy in high-risk UTUC patients [6,26]. However, current clinical guidelines (AUA, EAU, NCCN) continue to recommend neoadjuvant chemotherapy based primarily on data from MIBC, highlighting the need for additional evidence and treatment options specifically tailored to the adjuvant setting in UTUC [16,18].

Consistent with these findings, our study found that different types of adjuvant IO did not improve nor compromise survival outcomes, even after adjusting for demographic and pathological factors. There still remains a lack of powered evidence regarding the specific role of adjuvant IO therapies in UTUC, compared to MIUC. The role of IOs in UTUC treatment remains uncertain, and adjuvant chemotherapy appears to provide a more consistent prognostic and survival benefit for high-risk UTUC patients following surgery [26]. However, further research is necessary to fully explore all other treatment agents and combinations.

A key strength of this study is the detailed and comprehensive characterization of both demographic and pathological variables, which adds a level of real-world granularity often missing in previous studies. Our analysis includes a broad range of clinically relevant variables such as age, gender, BMI, ASA score, ECOG status, and comorbidities like type 2 diabetes mellitus and history of bladder cancer. Tumor-specific factors—including tumor size, location, multifocality, and margin status—alongside robust pathological variables such as TNM staging, grade, necrosis, and lymphovascular invasion, provide a multidimensional view of patient and disease profiles. This data allows for a more nuanced interpretation of outcomes and enhances the clinical relevance of our findings, distinguishing our work from prior literature that often relies on less detailed, registry-level inputs.

Multivariate Cox regression analysis identified pathologic nodal disease as the sole independent factor negatively impacting overall survival. This finding suggests that patients with nodal involvement (pN+) after nephroureterectomy are at a much higher risk of poor outcomes compared with those without nodal disease (pN0), highlighting the critical role that nodal status plays in predicting long-term survival. Hakimi et al. previously found that patients who underwent nephroureterectomy alone with positive lymph nodes had substantially worse 2-year OS and RFS compared to those with negative lymph nodes (42% vs. 86% for OS, 35% vs. 61% for RFS) [27]. Similarly, a study by Kagawa et al. also highlighted that patients with pN+ status had notably worse cancer-specific survival (CSS), RFS, and OS compared to those with pN0, even when receiving adjuvant IO [28]. Identifying pathologic nodal disease as an independent prognostic factor in our study further emphasizes the role of lymph node dissection in predicting outcomes for patients, while also highlighting the potential role of biomarkers—such as ctDNA—in identifying patients at highest risk for non-organ-confined disease [29,30]

While these findings may help inform treatment paradigms for UTUC, they are exploratory and should not define clinical practice standards. Further studies are warranted to validate the role of adjuvant IO in high-risk UTUC and ongoing trials may provide more definitive guidance.

These findings should be considered in the context of several limitations. The retrospective design excluded patients with unavailable survival status or unknown follow-up data, potentially introducing selection bias. Another limitation of the study is the relatively short median follow-up duration for the primary and secondary outcomes, 29 s. Given the natural progression of the disease, a longer follow-up period may provide a more accurate assessment of survival differences between groups. It is possible that, with extended follow-up, significant time-dependent differences in OS, URS, and NRFS may emerge. Future studies with longer observation periods may be worthwhile to better capture long-term survival trends and potential late recurrences. Additionally, the data may be prone to confounding effects, as not all variables may have been fully adjusted through PSM. The ROBUUST database also does not capture or account for detailed information on specific IO regimens, including their duration and doses. Patients within the cohort may have received suboptimal IO treatments, which could have obscured the benefit of the therapy. Given there is an absence of powered prospective trials for UTUC, further investigation through a robust PSM-driven retrospective study or randomized control trial (RCT) should be considered to understand the role that different IO types and durations may play in survival following nephroureterectomy for UTUC.

## 5. Conclusions

Using a large multi-institutional database for patients who underwent RNU, these findings suggest that adjuvant IO is not associated with improved oncologic outcomes of UTUC patients following extirpative surgery. Further consideration should be given to conducting randomized controlled trials and investigating the role of adjuvant immunotherapy in this subset of patients.

## Figures and Tables

**Table 1:** Demographic and Clinical Characteristics of UTUC Patients Among Treatment Groups.

	Before PSM			After PSM		
	Immunotherapy	No Adjuvant Therapy	*p*-Value	Immunotherapy	No Adjuvant Therapy	*p*-Value
N	75	1590	N/A	75	68	N/A
Age, median, (IQR), y	73 (67–79)	72 (65–79)	<0.001	73 (67–79)	73 (67.25–80)	0.686
Tumor Size, median, (IQR), cm	3.4 (2–4.6)	3 (2–5)	0.671	3.4 (2–4.62)	3.7 (2.15–6)	0.254
Sex, n (%)						
Male	49 (65.3)	932 (58.6)	0.227	49 (65.3)	40 (58.8)	0.423
Female	26 (34.7)	658 (41.4)		26 (34.7)	28 (41.2)	
N/A	0 (0)	0 (0)				
Histology, n (%)						
Urothelial	66 (97)	1465 (93)	0.26	66 (97)	63 (93)	0.47
Variant	2 (3)	69 (5)		2 (3)	4 (6)	
N/A	7 (0)	56 (0)		7 (0)	0 (0)	
Grade, n (%)						
Low	2 (2.7)	331 (22.3)	<0.001	2 (2.7)	3 (4.5)	0.547
High	73 (97.3)	1155 (77.7)		73 (97.3)	63 (95.5)	
N/A	0 (0)	104 (0)		0 (0)	0 (0)	
Pathologic T Stage (pT), n (%)						
*p* ≤ T2	22 (30.1)	992 (66.4)	<0.001	22 (30.1)	20 (29.4)	0.925
*p* > T2	51 (69.9)	501 (72.3)		51 (69.9)	48 (70.6)	
N/A	73 (0)	97 (0)		2 (0)	0 (0)	
Pathologic N Stage (pN), n (%)						
N0	26 (36.6)	472 (32)	<0.001	26 (36.6)	27 (39.7)	0.921
N+	19 (26.8)	123 (8.4)		19 (26.8)	18 (26.5)	
Nx	26 (36.6)	878 (59.6)		26 (36.6)	23 (33.8)	
N/A	4 (0)	117 (0)		4 (0)	0 (0)	
Neoadjuvant Therapy, n (%)						
Yes	37 (50.7)	161 (10.2)	<0.001	37 (50.7)	35 (51.5)	0.926
No	36 (49.3)	1423 (89.8)		36 (49.3)	33 (48.5)	
N/A	2 (0)	6 (0)		2 (0)	0 (0)	
Recurrence, n (%)						
Yes	34 (45.3)	463 (30.7)	<0.001	41 (54.7)	41 (63.1)	0.314
No	41 (54.7)	1045 (69.3)		34 (45.3)	24 (36.9)	
N/A	0 (0)	82 (0)		0 (0)	3 (0)	
Metastasis, n (%)						
Yes	41 (54.7)	1190 (86.1)	<0.001	41 (54.7)	20 (31.3)	0.006
No	34 (45.3)	192 (13.9)		34 (45.3)	44 (68.8)	
N/A	0 (0)	208 (0)		0 (0)	4 (0)	
Death, n (%)						
Yes	23 (30.7)	221 (14.7)	<0.001	23 (30.7)	13 (36.1)	0.136
No	52 (69.3)	1280 (85.3)		52 (69.3)	53 (80.3)	
N/A	0 (0)	89 (0)		0 (0)	2 (0)	

## Data Availability

The original contributions presented in this study are included in the article. Further inquiries can be directed to the corresponding author.

## Abbreviations

The following abbreviations are used in this manuscript:

IO	immunotherapy
UTUC	upper tract urothelial carcinoma
ROBUUST	ROBotic surgery for Upper tract Urothelial cancer STudy
URFS	urothelial recurrence-free survival
NFRS	non-urothelial-free survival
OS	overall survival
MIUC	muscle-invasive urothelial carcinoma
CSS	cancer-specific survival
RNU	radical nephroureterectomy (RNU)
NMIBC	non-muscle-invasive bladder cancer
MIBC	muscle-invasive bladder cancer
ADC	antibody–drug conjugate
BCG	Bacillus Calmette–Guérin
